# A New Oxyphospate for Crown Setting

**Published:** 1892-04

**Authors:** W. B. Ames

**Affiliations:** Chicago, Ills.


### A New Oxyphosphate for Crown Setting.

BY W. B. AMES, D.D.S., CHICAGO, ILLS.

Read before the Mississippi Valley Dental Association, March 9, 1892.

There are few, if any, processes in which chemistry is applied of which the knowledge is more empirical than of the various cement formations.

The best authors do not attempt to give any definite information on the various mortars and hydraulic cements, and Oxycloride of Zinc is dismissed with the statement that the ingredients form a cement much used by dentists in the filling of cavities of teeth. Of Oxy-phosphate of Zinc I have never been able to find a reference in any work on general chemistry.

Of the various oxy-phosphates offered for sale I am of the opinion, after examining a considerable number of them, that oxide of zinc is the basis and cement ingredient, other materials, such as silica alumina or magnesia in combination, acting only as modifiers of the plasticity before setting, and the hardness after setting, and do not enter into the crystallization.

After an extensive series of carefully recorded experiments I have arrived at the conclusion that a very limited number of the metallic oxides have the property of forming a cement in combination with phosphoric acid and water. The oxides of zinc, copper and mercury only have this property according to experiments.

The salts of mercury being so very potent for evil, as well as good, render the oxides of this metal undesirable as ingredients of cements to be used in the mouth, so that the only practical addition that I have been able to make through my experiments to the cement family, is the higher oxide of copper. Of copper oxides we have the cuprous or red, and the cupric or black. The red cuprous oxide of copper forms a cement with phosphoric acid and water that has good working qualities, but shrinks badly in setting, lacks strength, and becomes friable with crystallization.

The black cuprous oxide of copper forms with phosphoric acid and water, in proper proportions, a cement which has desirable working qualities, and a hardness and stability after crystallization which gives promise of its being a valuable addition to our list of materials for use in filling cavities, and the attachments of crowns and bridges. The most valuable property is the ability to use a large proportion of oxide in the mixture without hurrying the crystallization of the mass. With an unusually large proportion of oxide the crystallization is sufficiently slow to allow of thorough mixing and deliberate handling while filling a cavity, or setting a crown or bridge. It is peculiar in that it retains its plasticity for an unusually long time upon a cold glass slab, but crystallizes rapidly under the effects of the warmth of the body. While it gives plenty of time for manipulation, it hardens rapidly after the process has once commenced, and is harder in a few minutes after being placed in the mouth than is usual with oxy-phosphate of zinc. The crystallization of this cement, when the proper proportion of ingredients has been used, seems to be more perfect than any of the zinc oxide cements that I have ever seen. There is a flint-like hardness such as I have never met with in any other oxy-phosphates.

If a pure oxide is used, free of metallic copper, there is no staining of the tooth material from impregnation. If metallic copper be present there is a gradual discoloration, similar to that from impregnation of the tooth material from some amalgams.

There is, undoubtedly, a powerful antiseptic influence exerted by the cement while in the plastic state, as there is a small amount of phosphate of copper formed during the mixing, which is soluble in the free phosphoric acid of the plastic cement, but insoluble in the saliva after the crystalization has taken place. While the cement is hardening there is a distinct copper salt taste, which disappears when the crystallization is complete. This is analgous to the astringent acid taste from fresh oxy-phosphate of zinc fillings, which comes from the phosphate of zinc formed in the combination. Hot phosphoric acid will dissolve a considerable amount of the metallic oxides, and a small amount is always dissolved in the cold state. The practical application of this is, that the use of the oxide of a metal whose salts are highly antiseptic insures a considerable antiseptic influence from the cement. In as much as phosphoric acid attacks the more electro positive metals readily, it is naturally suggested that we should be careful about using an unplated steel spatula in the mixing of oxy-phosphates. If the phosphoric acid solution contains the proper proportion of water for best crystallization with the oxide used, this proportion may be modified to a detrimental extent by the combination of a portion of the acid with the metal of the spatula. A spatula of some of the more negative metals, or a steel spatula plated with negative metal is a more sensible instrument to use than one of steel only. The ivory spatula I would not consider as preferable to steel, but one of wood might in this connection be practicable, but for many reasons is inferior to thoroughly plated steel. A three inch druggist's spatula immersed in a strong solution of sulphate of copper, will become coated or plated with copper in such a manner as to make it a practical spatula for mixing this cement. This spatula should not be used for mixing any white cement as it is difficult to so thoroughly remove the black cement, that the other will not receive a decided tinge of the color. A glass slab from four to six inches square should be used.

---

DISCUSSION ON DR. AMES' PAPER.

Questions asked.

Q. Will it retain a polished surface?

A. It takes a glossy surface. Its main disadvantage is in the color, yet it does not stain the tooth.

Q. What other points in its favor?

A. It has the advantage of remaining plastic on a cold slab for a long time, giving plenty of time to the operator, in fact, my assistant often has the cement ready before I begin to work; after it gets the influence of the warmth of the mouth, it begins to crystallize and sets quickly.

Q. Is there any difference in mixing it?

A. Make it stiff.

Q. Does it mix as hard as cement?

A. About the same. You can mix it stiffer because it does not set so quickly on the slab. If mixed thin it does not set as hard.

Q. Is it adhesive?

A. It is very adhesive.

Q. Is it an irritant?

A. Not more so than Oxyphosphate of Zinc, as the only irritant is the phosphoric acid used.

*Dr. Cassidy.*--It seems to me that this material is not a true chemical compound.

*Dr. Ames*--I do not profess to say anything about the chemical part; it is my opinion that the phosphate of copper on account of its antiseptic properties should have the preference.

*Dr. Heise.*--Is this acted upon by the saliva, and does it stain the tooth?

*Dr. Ames.*--It is insoluble in the saliva. If the pure black oxide of copper is used there is no discoloration. I make use of it for filling children's teeth. Of course the black shows through the thin walls of the enamel, but where the pure oxide is used it does not discolor the tooth.

*Dr. Morrison.* Have you experimented or produced any mixture to get rid of the color? For instance, mixing both oxide of copper and oxide of zinc with the phosphoric acid.

A. The difficulty is that each oxide requires a different consistency of phosphoric acid,but this might be found out by experience. This cement requires a larger proportion of water than any other I have ever seen, and this indicates that we obtain more perfect crystallization and consequently a more enduring filling. We would not get the best working qualities of either if used in combination, on account of the difference of specific gravity and crystallization of the phosphoric acid.

*Dr. Morrison*--I have had trouble with ordinary cement getting hard while being mixed. What is the cause of this?

*Dr. Ames.*--Too much water in the acid.

*Dr. Smith.*—Dr. Jay has asked the question as to what cement is the best. Who knows much about it? A certain doctor told me this last summer that he made very enduring cement fillings, and I asked him how he did it, he said by taking care of the material, and through this precaution he obtained the best results. I think, however, that Dr. Taft put the matter about right when he once said that "the best oxyphosphate filling is only a poor filling." We deceive our patients unless we tell them the true character of this filling. I would like to know of a good cement.

*Dr. Wright.*—Dr. Smith's remarks are rather discouraging; it seems to me that Dr. Ames has made a step in advance in this direction.

*Dr. Smith.*—It is a mistake to think that I was saying anything against Dr. Ames. His material is new, and I have not yet tested it. I have great respect for Dr. Ames' ability, and admire the interest he has manifested in this direction, and hope that he will yet discover some combination that will make a permanent filling of good color.

*Dr. Ames.*—I look forward to great improvements in cement fillings over anything we now have; there is a very great difference in what I have found in the results obtained. I have used this cement with better results than I have ever obtained from any other. It makes a much more enduring filling than oxyphosphate of zinc; where the phosphate fillings have stood only six months, fillings of this cement in the same mouth have stood for one and a half years. It withstands mastication better than oxyphosphate and has better edge strength. Its greatest advantage is in setting crowns, bridges, etc.

